# Singing in Churches

**Published:** 1871-04

**Authors:** 


### SINGING IN CHURCHES.

I WENT into a meeting-house in Fifth Avenue, New York, not long ago. Both the outside and inside of the building indicated substance, stateliness, and power. Having some knowledge of New Yorkers, it was evident at a glance that there were men of great wealth there; in truth, within a circuit of twenty feet there were representatives of more than fifteen millions of dollars; and when the plates went round for the "offerings" of the charitable, they were returned, and, on being emptied, there was counted out twenty-three hundred dollars. The minister was a man of might; at times his eloquence was soul-thrilling, and he seemed carried away with his own burning thoughts; his illustrations were to the point, and made every subject as plain as the day. Distinct and measured in his enunciation, classical in his language, commanding in his method, and strong in his thoughts, he held the whole assembly, and at the end of a sentence you could hear a pin drop, or you couldn't; don't remember which just now.

The music of the quartette in the choir was beautiful, charming, superb, and made me as mad as a March hare, because I couldn't join in; three tunes were sung, but it seemed as if not a single solitary soul had ever heard one of them before, except one man, who did try to take a hand in the last one, but he didn't try long, and gave up in despair.

There was one thing in the arrangement about the choir which was commendable; there were no curtains to hide them from the people, so they might eat pea-nuts, and pass love-messages in pencil mark when the minister was not interesting.

There was rather an oddity in one of the musical performances. The lady began right away, and sang half a line before the tall fellow beside her got fairly awake, and instead of joining in with her where she was, he seemed to think it was his duty to pronounce all the words from the beginning of the line, and the way he galloped along was really amusing; but the woman was very considerate, for as soon as she noticed that he was doing his best to catch up with her, she "slowed" like, until, puffing and blowing like all the world, he caught up with her at last. It is hard to tell what had taken possession of them; but the queerness of the whole thing was increased, for it really seemed a kind of fun to them, and they repeated the operation at the beginning of every verse. The effect, however, was agreeable rather than otherwise, so that at the end there was quite a temptation to "encore" the whole performance, up to that point, but no farther; for the whole assembly rose worshipfully to their feet, to sing the doxology, but they didn't, because nobody took the lead, and silence reigned profound. On glancing at the choir, it was observed that the young lady whose place it was to begin was otherwise engaged; something had set her a giggling, instead of singing, and like the man with a cork leg, she couldn't stop; presently the minister turned his eyes in that direction and saw what was going on; it seemed as if he looked forty thunders; the tall man at the lady's side, as if he thought the situation was becoming desperate, gave her a tremendous poke in the ribs; this brought her to herself, and the doxology went on. The instant the last word of it was sung, the lady fell helplessly in her seat, and turning her pretty head aside, completed the interrupted titter, but it took her quite a while to do so.

The doxology was sung to grand "Old Hundred," and the people, as if oppressed with the long pent-up music of the soul, burst forth in one of those deep, and loud, and solemn utterances of devotion, which seemed almost to raise the roof from the building, showing at once that music is the medium of devotion; that it is an instinct, a help to heavenly worship, greater by far when the worshipper joins in than if only a hearer. Then comes the practical question, Is it not a robbery for choirs to sing tunes about which the people know nothing, and thus prevent them from enjoying that natural expression of worship and devotion which is connected with that part of the services of the sanctuary?

It is certainly proper and right that new tunes should be introduced from time to time, else there would be an end of progress; but the people should be made acquainted with them by degrees at minor meetings first, and only introduce them into the great congregation after the stand-bys of the prayer-meeting, the lecture-room, and the Sunday-school have thoroughly mastered them. A new tune is more easily and more quickly learned if half an assembly joins in, than from the singing of half a dozen persons in the choir.

A familiar tune sung to familiar words increases the pleasure and the enjoyment and the Christian feeling of the singer tenfold. Persons of any, the least observation, have seen and felt this many times in the past. But let a hymn be given out which had been always associated with a particular tune, and when the people rise to its performance, ready to burst forth on the instant in the old familiar strain, let the choir open upon them some new jig, fresh from the theatre, what a fall is there, what a deep and universal disappointment there is, and how angrily do the people stand mute and indignant!

Music is emotional; it is prayer, and praise, and worship, all in one. To sing from the heart, feeling the sentiment the words express, is the very sweetest form of divine worship. It is truth, and doctrine, and humility, and thanksgiving, all in one. And when it is so much to the Christian heart, such a great aid to worship; when it is the passage-way of the soul, from heart to heaven, surely everything possible should be done to encourage and enable every hearer in a religious assembly to "join in," and perhaps there is no quicker, no easier, no more efficient way to have every man, woman, and child sing in a religious assembly, than to have fewer tunes, fewer hymns, and let each hymn have its characteristic tune. A great part of the enthusiasm of large assemblies in singing a national hymn is the universality with which it is sung. With what tremendous effect is a chorus sometimes sung when every human being seems to join in! Depend upon it, music is a greater quickener of religious thought, of heavenly emotion, than preaching or prayer and all other instrumentalities combined. A main reason why the Methodists succeed in getting large numbers into their places of meeting may be the deep earnestness with which every man, woman, and child seems to join in the songs of praise. From personal observation, extending through many years and over a large section of our country, there is more deep enjoyment in the whole-souled singing of the negro congregations in the South, in one Sunday, than there is in every Northern church put together in a whole year.

Whenever a strange tune is sung to very familiar words, there is a feeling similar to that experienced by a slight change in the last line of one of Watts's familiar verses: —

> "False are the men of high degree,
> The baser sort are vanity;
> Laid in a balance both appear,
> Just like a" — barrel full of beer.

The lesson is, as the love of music is, almost universal, and man is a religious animal; a good way to entice the multitude into our churches on Sundays is to have good music; and to have it good, let familiar tunes be sung to familiar words, and let it be considered the religious duty of every soul to participate, to join in the song.

